# State sanctuary policies and the immigration enforcement activities across US counties: implications for population health

**DOI:** 10.1093/haschl/qxag212

**Published:** 2026-08-14

**Authors:** Alexandra Eastus, Caroline Kravitz, Alina Schnake-Mahl, Tamara Rushovich, Serenity Lita Lillibridge, Brent Langellier

**Affiliations:** Department of Health Management and Policy, Dornsife School of Public Health, Drexel University, Philadelphia, PA 19104, United States; Department of Health Management and Policy, Dornsife School of Public Health, Drexel University, Philadelphia, PA 19104, United States; Department of Health Management and Policy, Dornsife School of Public Health, Drexel University, Philadelphia, PA 19104, United States; Urban Health Collaborative, Dornsife School of Public Health, Drexel University, Philadelphia, PA 19104, United States; Urban Health Collaborative, Dornsife School of Public Health, Drexel University, Philadelphia, PA 19104, United States; Urban Health Collaborative, Dornsife School of Public Health, Drexel University, Philadelphia, PA 19104, United States; Department of Health Management and Policy, Dornsife School of Public Health, Drexel University, Philadelphia, PA 19104, United States; Urban Health Collaborative, Dornsife School of Public Health, Drexel University, Philadelphia, PA 19104, United States

**Keywords:** immigration policy, health equity, immigration enforcement, safety-net programs, social determinants of health

## Abstract

**Introduction:**

Recent increases in US immigration enforcement may contribute to an anti-immigrant environment that poses serious health and social risks. In response, several states have enacted sanctuary policies limiting cooperation with federal immigration authorities, though their population-level impacts remain unclear. This study estimated associations between state sanctuary policy enactment and county-level public program participation by baseline immigration enforcement.

**Methods:**

County-level data from 2011 to 2023 were obtained from the American Community Survey and Syracuse Transactional Records Access Clearinghouse. Outcomes included county-level Medicaid and Supplemental Nutrition Assistance Program (SNAP) participation. Sanctuary policies were modeled as a time-varying exposure using a staggered difference-in-differences framework, with analyses stratified by baseline immigration enforcement.

**Results:**

We found no statistically significant association between sanctuary policy enactment and Medicaid or SNAP enrollment per 10 000 likely eligible individuals in counties with high baseline enforcement; however, estimated percent changes were positive (Medicaid percent change: 28.6, 95% CI: −14.9, 94.3; SNAP: 9.9, 95% CI: −81.6, 557.5). In counties with low baseline enforcement, associations were not statistically significant, and estimates were smaller in magnitude.

**Conclusion:**

These findings suggest that protective immigration policies may partially mitigate chilling effects and promote engagement with social supports among eligible populations. However, estimates should be interpreted cautiously given the limited number of treated states.

## Introduction

HR1—the One Big Beautiful Bill Act—includes approximately $170 billion in new funding for US Immigration and Customs Enforcement (ICE), the division of Homeland Security responsible for enforcing federal immigration laws within the US interior.^1^ This dramatic increase in funding will make the ICE budget larger than military budgets in all countries other than the United States, Russia, and China.^2^ Although enforcement efforts have been framed as targeting individuals with serious criminal convictions, emerging evidence suggests that 70% of individuals detained by ICE have no criminal convictions.^3^

Immigration enforcement policies and practices can shape population health through multiple pathways. One common mechanism is the use of detainer requests, in which federal authorities ask local law enforcement agencies to hold individuals beyond their scheduled release to facilitate transfer to federal custody.^4^ Detainer requests are a widely used proxy for local immigration enforcement intensity and have been linked to reduced healthcare utilization^5^ and reduced public program participation,^6^ often through “chilling effects” driven by fear, uncertainty, and reduced institutional trust.^7,8^ In the context of immigration enforcement, chilling effects refer to the avoidance or underutilization of healthcare services and public benefits out of fear of exposing oneself or members of their social networks to immigration enforcement.^7,8^ Importantly, these effects extend beyond individuals directly targeted by immigration enforcement. Many immigrant families are mixed-status households that include both citizen and non-citizen members. As a result, concerns regarding immigration enforcement may discourage healthcare utilization,^7^ benefit participation,^8^ and civic engagement^9^ even among individuals who are themselves eligible for services. Medicaid and Supplemental Nutrition Assistance Program (SNAP) participation are especially relevant outcomes because enrollment requires interaction with government agencies and disclosure of personal information, making these programs susceptible to enforcement-related chilling effects among eligible individuals, including but not limited to people in mixed-status households.

In response to these dynamics, several states and localities have enacted sanctuary policies that limit cooperation between local law enforcement and federal immigration authorities, including restrictions on honoring detainer requests or sharing information with ICE. By reducing cooperation with federal immigration authorities and signaling protection,^8,10^ sanctuary policies may improve health by decreasing stress,^11^ fostering trust in public institutions, and facilitating engagement with healthcare services and safety-net programs.^6,12^ Further, immigrant-inclusive policies may also shape behavior by influencing perceptions of government responsiveness, thereby impacting interactions with public institutions.^13^

Despite these hypothesized benefits, evidence on the health and social impacts of sanctuary policies remains limited and mixed. Much of the existing literature examines broader “pro-immigrant” policy environments—such as composite indices of inclusivity—rather than identifying the effects of individual policies.^14,15^ Among studies that focused explicitly on sanctuary policies, definitions vary widely (eg, inclusion of local vs state policies, differing legal criteria), limiting comparability across studies and obscuring which policy components may drive observed effects.^6,12,16^ In addition, many prior analyses relied on cross-sectional designs, limiting causal inference. As a result, it remains unclear whether sanctuary policies themselves have measurable population-level effects and whether these effects vary by the underlying intensity of local immigration enforcement. The effects of sanctuary policies may vary by local immigration enforcement context because these policies are designed to limit cooperation between local jurisdictions and federal immigration authorities. In counties with historically high levels of immigration enforcement, state sanctuary policies may have larger effects because these communities experience greater exposure to enforcement-related risks and chilling effects. In these settings, sanctuary policies may represent a more substantial change to the local policy environment by reducing cooperation with federal immigration authorities in communities where enforcement activities have been more visible or prevalent. Consequently, sanctuary policies may be more likely to reduce enforcement-related chilling effects and increase engagement with public institutions. In contrast, sanctuary policies may have smaller effects in low-enforcement settings where perceived risks associated with enforcement are already lower and local cooperation with federal immigration authorities may be less common, resulting in less opportunity for the policy to change local enforcement behavior.

Recent work demonstrated that higher levels of local immigration enforcement were associated with lower Medicaid and SNAP enrollment among likely eligible adults.^8^ Building on these findings, the present study evaluates whether state sanctuary policy enactment—a policy intended to limit cooperation with federal immigration enforcement—is associated with changes in public program participation. To address these gaps, we use a staggered difference-in-differences (DiD) analysis to estimate the impact of state sanctuary policy enactment (2013-2021) on county-level safety-net program participation among likely eligible populations. In secondary analyses, we examine associations with voter participation. Unlike prior observational studies of immigration enforcement, this quasi-experimental study leverages staggered variation in the timing of policy enactment to estimate changes in public program participation. We compile a novel dataset of state sanctuary policies using consistent legal definitions applied across time and link these data to county-level outcomes from 2011 to 2023.^17^ This period captures substantial variation in federal enforcement priorities and state policy responses. Finally, we assess whether associations differ by baseline levels of immigration enforcement, providing insight into how the local enforcement context may modify the effects of state sanctuary policies.

## Methods

### Measures

#### Exposure

The primary exposure was the enactment of a state sanctuary policy, defined as a state law enacted between 2009 and 2021 that limits cooperation with federal immigration enforcement (Appendix A).^17-20^ We identified state sanctuary policies by systematically searching the National Conference of State Legislatures (NCSL) Immigration Database, a collection of enacted state-level immigration laws from 2008 to present. We reviewed and coded each law to ensure inclusion criteria were met. We included state sanctuary policies if they were enacted between 2013 and 2021 and specifically limited local law enforcement agencies from participating in federal immigration enforcement activities.^19,20^ County-year observations were categorized as “treated” beginning in the first year a state sanctuary policy was enacted (2013-2021) and remained treated thereafter. Counties in states that never enacted a state sanctuary policy during the study period served as the comparison group to avoid potential bias from anticipation effects among eventually treated counties. We excluded Rhode Island from analyses because the state enacted a sanctuary policy before the study period. We were unable to account for policy end dates or litigation-related enjoinments—court-issued injunctions that halt or prohibit specific behaviors or policies while a lawsuit is pending—but after searching online media sources and court documents, found no indication that any of the state policies were challenged, enjoined, or repealed during the study period. See Appendix Figure S1 for details on the inclusion of sanctuary policies.

#### Effect heterogeneity

We assessed effect heterogeneity by baseline enforcement using county-level ICE detainer requests issued per 10 000 non-citizens.^21^ Data were obtained from ICE by the Syracuse University Transactional Records Access Clearinghouse via Freedom of Information Act requests.^21^ We classified counties based on baseline (2011) enforcement levels as above or below the national median. This approach captures differences in local enforcement intensity, for which detainer requests serve as a well-established proxy.^22^ We hypothesized that sanctuary policies would have larger effects in high-enforcement counties because these communities experience greater exposure to immigration enforcement and therefore greater potential for enforcement-related chilling effects.

#### Outcomes

Primary outcomes were Medicaid and SNAP participation, measured as the number of enrollees in a county per 10 000 likely eligible residents. The denominator reflects residents below 200% of the Federal Poverty Level (FPL).^23^ County-level data on program participation were obtained from the American Community Survey (ACS) 5-year estimates (2011-2023). The ACS 5-year estimates represent rolling averages that maximize county-level coverage. Consequently, estimated policy effects should be interpreted as changes in average county-level participation over rolling 5-year periods rather than discrete annual changes.

We tested registered voter participation as a secondary outcome, measured as the percentage of eligible registered voters in a county who turned out in even-year state and federal elections. Voter turnout data were collected from the National Neighborhood Data Archive, derived from the Election Administration and Survey datasets.^24^

#### Covariates

Models were adjusted for theoretically relevant county- and state-level covariates associated with both state sanctuary policy adoption and social outcomes. County-level covariates included the baseline proportion of non-citizens and labor force participation derived from ACS 5-year estimates. To account for local policies that could influence sanctuary policy enactment and public program uptake, we included an indicator for whether the county ever had a 287(g) agreement, reflecting the potential anticipatory and residual effects of federal immigration enforcement partnerships. 287(g) agreements deputize local law enforcement officials to carry out immigration-related tasks, expanding federal immigration authority within communities. At the state level, we adjusted for the political party of the state governor as a time-varying measure of state partisan composition from the NCSL.^25^

We explored adjustment for additional time-varying state and county immigrant-related policies, including state Medicaid eligibility within the 5-year lawful permanent resident ban, state Medicaid expansion status, and local sanctuary policy status. State Medicaid eligibility within the 5-year ban and Medicaid expansion status were not included because of correlation and convergence concerns. Sensitivity analyses adjusted for county sanctuary policy status. Voter participation models additionally controlled for presidential vs midterm election years to adjust for the inherent participation differences.

### Study sample

We created 6 analytic samples (2 per outcome) due to differing data sources and stratification by baseline detainer requests. Our sample includes all county-year observations from 2011 to 2023, with the exception of Alaska for analyses of voting outcomes. For treatment, we included all state sanctuary policies meeting our inclusion definition that were enacted between 2013 and 2021 in our sanctuary dataset. We included 2 pre- and post-policy years in our study period to estimate pre-post trends. We excluded any county-years with missing outcome data. Medicaid data were unavailable in 2011 (analyses covered 2012-2023); voting analyses excluded Alaska due to data limitations; and all analyses excluded Rhode Island since the state policy was enacted prior to the study period.

### Statistical analysis

We first compared sociodemographic characteristics between ever-treated and comparison counties. We then estimated the association between state sanctuary policy enactment and outcomes using a staggered DiD approach with the Callaway and Sant’Anna estimator (R package *did*).^26^ This method accounts for variation in policy adoption timing across states and provides group-time-specific and aggregated average treatment effects of the treated (ATT). In this study, the ATT reflects the difference-in-differences in each outcome for treated counties relative to never-treated counties (first difference) before vs after policy enactment (second difference), within the same enforcement strata.

All models included a standardized baseline propensity score as a time-invariant covariate to adjust for pretreatment differences in the likelihood of ever being treated. We used linear models with each outcome as the log-transformed rate of enrollment per 10 000 likely eligible people (people under 200% FPL). Standard errors were estimated using nonparametric bootstrapping clustered at the state level, consistent with the default settings in the *did* package. For primary results, we report dynamic group-time ATT estimates, expressed as prevalence ratios by exponentiating logged outcome models. All analyses were conducted separately within high and low baseline enforcement strata; for example, treated counties with high baseline immigration enforcement were compared only to never-treated counties that also exhibited high enforcement.

**Figure 1.**
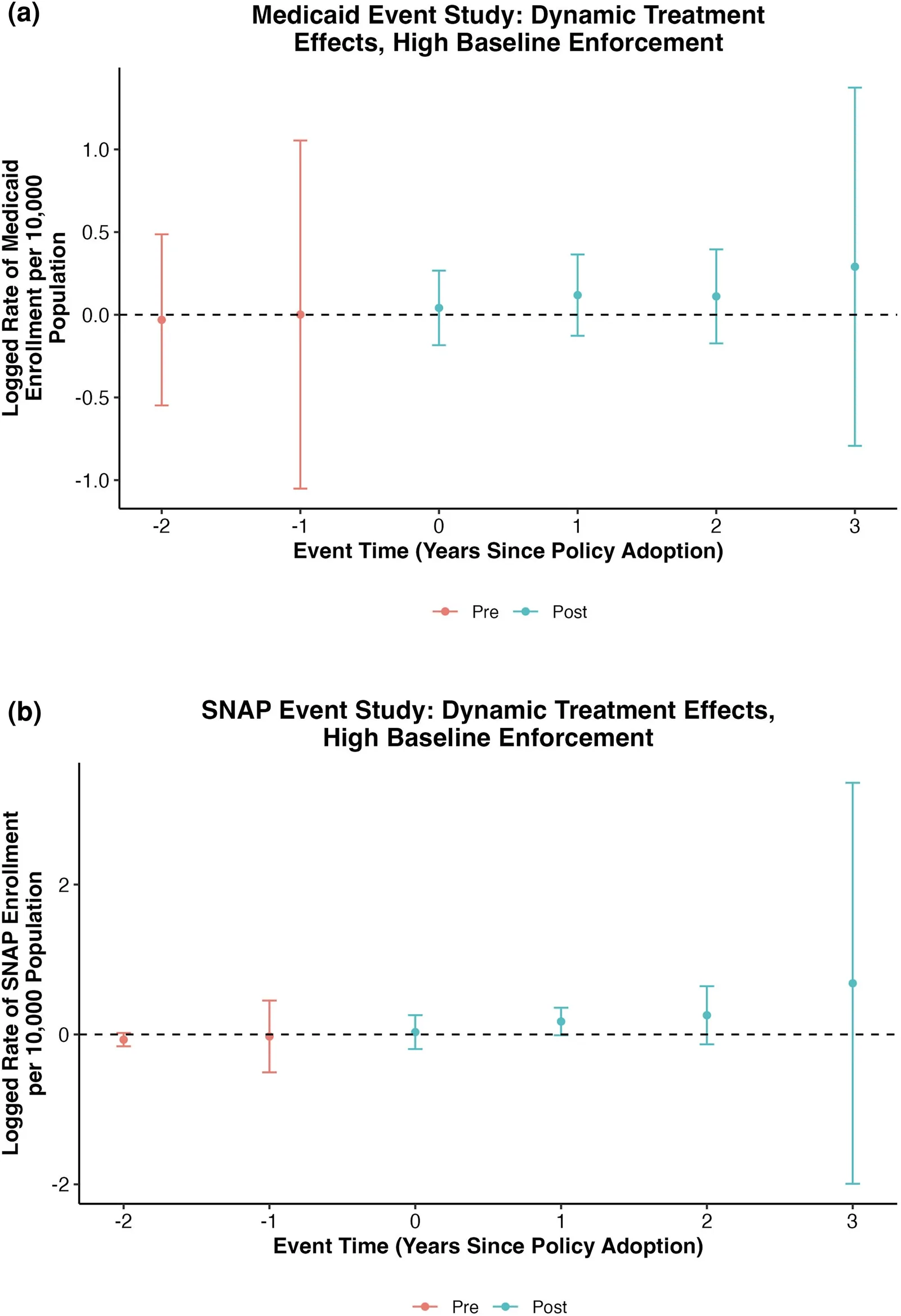
Conditional dynamic event study plots, high immigration enforcement samples only: (A) Medicaid; (B) SNAP. Notes: Event study plots adjusted for the following baseline indicators: (1) county population proportion of non-citizens, (2) a binary indicator of whether the county ever enacted a 287(g) agreement, and (3) governor's political party affiliation. Immigration enforcement quantified at baseline (2011)—low enforcement ≤ median number of detainer requests per 10 000 non-citizens; high enforcement > median number of detainer requests per 10 000 non-citizens. Medicaid program participation: Estimated by the number of individuals participating in Medicaid divided by the total number of individuals in poverty (less than or equal to 200% FPL) per county and year (per 10 000 likely eligible low-income individuals). Supplemental Nutrition Assistance Program (SNAP) participation: Estimated by the number of households participating in SNAP divided by the total number of low-income households per county and year (per 10 000 households). Fully adjusted models included (1) time-varying covariates, including county-level proportion of non-citizens, labor force participation, and state governor party affiliation; and (2) a time-invariant covariate, indicating whether county ever had a 287(g) agreement. Note: all Medicaid models only had 1 year in the pretreatment period due to data restrictions in county-level Medicaid participation.

We verified key assumptions underlying the staggered DiD design. First, we tested for conditional parallel pre-policy trends between treated and comparison counties (Appendix Figure S2). Second, we evaluated whether there were anticipatory effects in periods preceding the enactment of sanctuary policies. Both assumptions were visually examined using event-study plots of propensity score-adjusted estimates from the Callaway and Sant’anna model. These plots showed no statistically significant differences in pretreatment trends between groups, supporting the parallel trends and anticipatory effects assumptions (Figure 1: high-enforcement counties; low enforcement in Appendix Figure S3). Fully adjusted models additionally controlled for the county- and state-level covariates described above.

### Sensitivity analysis

We re-ran all models excluding Virginia and Utah as each enacted both anti-sanctuary (eg, legislation that promotes or requires cooperation with federal immigration authorities) and sanctuary policies during the study period, which may bias the average estimate toward the null for the treated groups. In additional sensitivity analyses, we used not-yet-treated counties as an alternative comparison group to test robustness of our findings. Finally, we re-estimated the primary stratified models with adjustment for county-level sanctuary policy status and estimated overall models that additionally adjusted for time-varying immigration enforcement and county-level sanctuary policy status.

This study used publicly available, deidentified data that did not require Institutional Review Board review. We followed the Strengthening Reporting of Observational Studies in Epidemiology reporting guidelines. We used R version 4.2.3 (2023-03-15) for data cleaning and analyses.

### Limitations

Our study had several limitations. First, results may be subject to unmeasured confounding from concurrent time-varying factors. States that adopt sanctuary policies may simultaneously implement other changes. While we adjusted for key covariates and baseline propensity to adopt sanctuary policies, residual confounding from unmeasured state- and county-level factors remains possible. Although we estimated sensitivity analyses adjusting for time-varying immigration enforcement and county-level sanctuary policy status, these variables were not included in primary models because our primary analyses were designed to evaluate effect heterogeneity by baseline enforcement. As a result, residual confounding related to local immigration policy environments cannot be excluded.

Second, the use of county-level data limits our ability to assess individual- or household-level responses and masks heterogeneity by immigration status, nativity, and race/ethnicity. County-level estimates should not be interpreted as evidence of individual-level behavioral change and may obscure important differences across immigrant, mixed-status, and US-born populations.

Third, treatment was defined based on state sanctuary policy enactment and did not account for local sanctuary policies operating within states that lacked statewide sanctuary protections. Consequently, some counties classified as untreated in primary analyses may have been exposed to local sanctuary policies, potentially resulting in exposure misclassification. However, sensitivity analyses incorporating time-varying county-level sanctuary policy status yielded directionally consistent findings, suggesting that any bias was likely limited and toward the null. Nonetheless, because county-level sanctuary policy status could not be incorporated into the primary doubly robust stratified models, some exposure misclassification may remain.

Fourth, we were unable to account for policy end dates, temporary suspensions, or litigation-related enjoinments, which may result in exposure misclassification. However, we do not expect this to meaningfully impact our findings because prior research shows that immigration policies can impact health even when their legal status is uncertain or unenforced, as fear and perceived risk may persist even after policy changes.^27^

Finally, Medicaid and SNAP participation were measured using ACS 5-year estimates, which are rolling averages that share underlying observations across consecutive years. As a result, short-term changes in program participation may be smoothed, and estimated policy effects should be interpreted as reflecting changes in average county-level participation over rolling 5-year periods, rather than discrete annual changes immediately following policy enactment. We used ACS 5-year estimates to retain national coverage of US counties, as 1-year estimates are unavailable or unreliable for many smaller counties. Future studies using individual-level longitudinal data are needed to isolate mechanisms, identify heterogenous effects across populations, and assess the independent and combined effects of local sanctuary policies and state sanctuary policies on health and social outcomes.

## Results

### Sample characteristics

**Figure 2.**
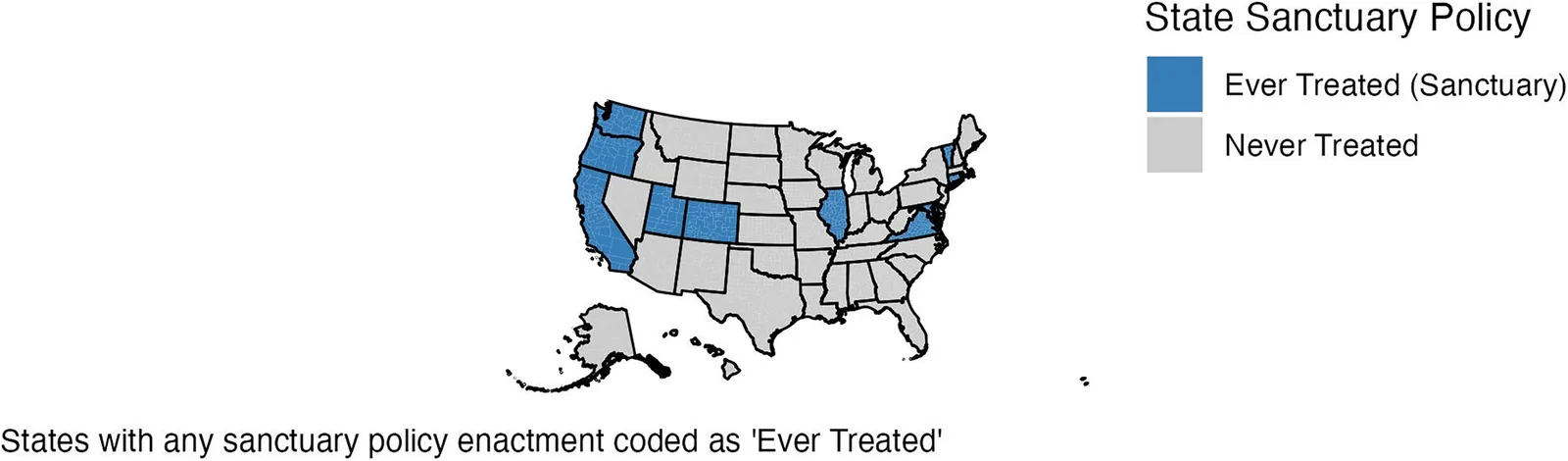
US counties by state sanctuary policy status (2013-2021). Notes: States with any sanctuary policy enactment between 2013 and 2021 coded as “ever treated.” Rhode Island enacted a sanctuary policy but was excluded in analyses due to the policy being enacted in the study pre-period (2011).

Between 2013 and 2023, 568 counties in 12 states (representing 92.5 million people or 28% of the US population) were “treated” with state sanctuary policies, and 2744 counties in 38 states were untreated (Figure 2).

At baseline (2011 for SNAP; 2012 for Medicaid), 1329 never-treated and 194 ever-treated counties had low immigration enforcement (< median detainer requests per 10 000 non-citizens), whereas 1327 never-treated and 195 ever-treated counties had high immigration enforcement (> median).

Counties in states that never enacted a sanctuary policy were more likely to have a 287(g) agreement, have a higher proportion of non-Hispanic White residents, and have Republican governors than counties in states that ever enacted a sanctuary policy (Table 1). Among counties with high enforcement in non-sanctuary states, the average number of 287(g) agreements and detainer requests per 10 000 non-citizens was substantially greater than in high-enforcement counties in sanctuary states and was greater than in low-enforcement counties overall (Table 1). State policies were generally enacted in 3 time periods: 3 states enacted a policy between 2013 and 2014 (ie, early adopters), 4 between 2015 and 2018 (ie, middle adopters), and 3 between 2020 and 2021 (ie, late adopters).

**Table 1.** Baseline characteristics of US counties in states that did and did not enact sanctuary policies by baseline immigration enforcement, 2012-2023.

	US counties—baseline low immigration enforcement^a^		US counties—baseline high immigration enforcement^a^	
	Never sanctuary state	Ever sanctuary state	Never sanctuary state	Ever sanctuary state
	*N* = 1329	*N* = 194	*N* = 1327	*N* = 164
**Treatment definition**	Counties in states that never enacted a sanctuary policy	Counties in states that enacted a sanctuary policy	Counties in states that never enacted a sanctuary policy	Counties in states that enacted a sanctuary policy
**Characteristic**				
**Primary outcome of interest (%)** ^b^				
Proportion of Medicaid enrollees per 10 000 low-income individuals (<200% FPL)	17.6 (13.1, 22.7)	14.44 (10.2, 19.9)	17.5 (14.1, 21.8)	16.4 (12.0, 21.3)
Proportion of SNAP participants out of likely eligible low-income population (<200% FPL)	43.5 (32.8, 52.1)	41.4 (29.0, 49.0)	44.0 (36.3, 52.1)	35.4 (25.0, 46.0)
Registered voter turnout	50.6 (45.0, 57.2)	47.9 (42.6, 53.4)	49.4 (43.7, 55.6)	56.7 (50.9, 64.6)
**Immigration enforcement activity—median (IQR)** ^b^				
County-level detainer requests issued per 10 000 non-citizens	27.0 (7.7, 333.3)	6.0 (1.4, 19.1)	23427.9 (13280.4, 47279.1)	22392.0 (12309.0, 35008.0)
**County-level covariates** ^b^				
Population proportion, non-citizen individuals (%)	0.9 (0.4, 2.1)	1.4 (0.8, 3.6)	2.1 (0.9, 4.1)	3.0 (1.3, 8.0)
Population proportion, individuals with income less than 200% FPL (%)	15.7 (11.5, 20.5)	12.8 (8.9, 18.2)	16.2 (12.4, 20.1)	13.5 (10.0, 17.0)
Population proportion, Hispanic (%)	2.1 (1.1, 4.3)	3.0 (1.3, 6.1)	4.7 (2.2, 10.4)	8.7 (2.8, 20.7)
Population proportion, non-Hispanic, American Indian Alaskan Native (%)	0.3 (0.1, 0.8)	0.1 (0.1, 0.3)	0.3 (0.1, 0.6)	0.3 (0.1, 0.6)
Population proportion, non-Hispanic Asian (%)	0.4 (0.1, 0.8)	0.6 (0.3, 2.3)	0.6 (0.3, 1.2)	1.0 (0.5, 3.1)
Population proportion, non-Hispanic Black (%)	0.9 (0.3, 4.2)	5.8 (1.1, 19.2)	3.3 (0.7, 14.3)	2.8 (0.8, 8.7)
Population proportion, non-Hispanic White (%)	9.18 (7.22, 9.58)	81.8 (63.8, 93.7)	81.8 (64.7, 91.3)	77.9 (61.5, 88.0)
County ever had a 287(g) agreement^c^	23 (2.3%)	3 (1.8%)	117 (8.1%)	5 (2.4%)
**State-level covariate**				
State governor Democrat political affiliation	362.0 (35.5%)	73.0 (42.9%)	378.0 (26.1%)	175.0 (84.1%)
State governor Republican political affiliation	657.0 (64.5%)	97.0 (57.1%)	1069.0 (73.9%)	33.0 (15.9%)

Baseline is 2012 for Medicaid and 2011 for all other outcomes.

Abbreviation: FPL, Federal Poverty Level.

^a^Immigration enforcement quantified as the number of detainer requests per 10 000 non-citizens. Baseline enforcement categorized as high if greater than the national median detainer requests for 2011 (2012 for Medicaid) and low if less than or equal to the median.

^b^Median (IQR).

^c^ *n* (%) (*n* = counties).

### Staggered difference-in-difference estimates

**Figure 3.**
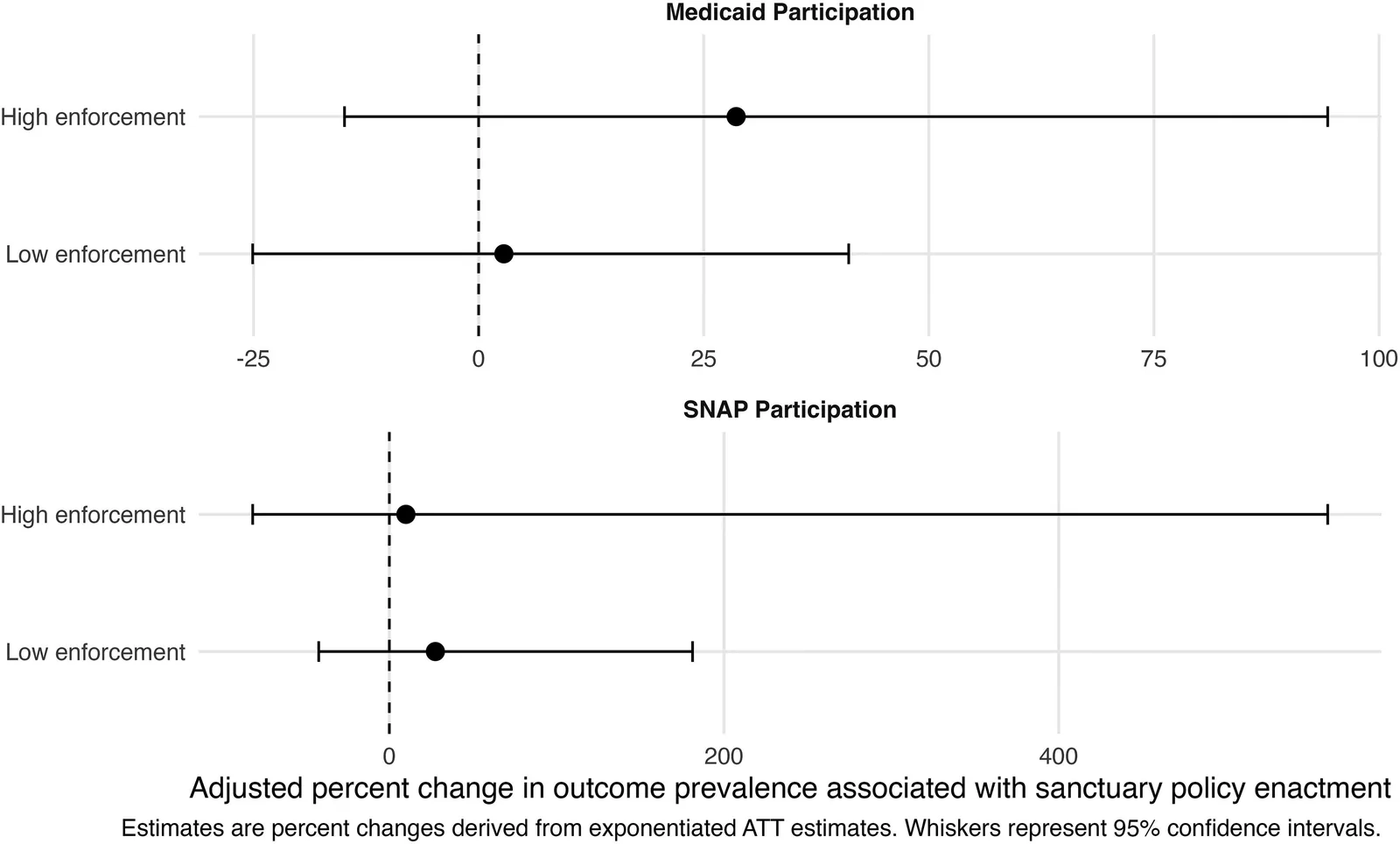
Adjusted percent change in Medicaid and SNAP participation associated with state sanctuary policy enactment, stratified by baseline immigration enforcement. Notes: Percent changes are derived from exponentiated average treatment effect on the treated (ATT) estimates from the staggered difference-in-difference models. Whiskers represent 95% confidence intervals. Baseline immigration enforcement was defined using the median number of Immigration and Customs Enforcement (ICE) detainer requests per 10 000 non-citizens.

In the fully adjusted models, state sanctuary policy enactment was not significantly associated with public program participation (Appendix Table S1). However, estimated effects were consistently positive, particularly in counties with high baseline immigration enforcement. Medicaid enrollment per 10 000 likely eligible population was not significantly different following state sanctuary policy enactment, although the estimated percent change was 29% (percent change in prevalence: 28.6; 95% CI, −14.9, 94.3; Figure 3) in counties with high baseline immigration enforcement. In contrast, the estimated percent change in Medicaid enrollment was 3% in low-enforcement counties (percent change in prevalence: 2.8; 95% CI, −25.1, 41.1; Figure 3). The estimated percent change in SNAP enrollment per 10 000 likely eligible population was 10% (percent change in prevalence: 9.9; 95% CI, −81.6, 557.5; Figure 3) among counties with high baseline immigration enforcement. Additionally, the estimated percent change in SNAP enrollment per 10 000 likely eligible population was 27% (percent change in prevalence: 27.5; 95% CI: −42.2, 181.2) among counties with low baseline enforcement. Although none of the estimated changes in Medicaid or SNAP enrollment reached statistical significance, all point estimates were positive and should therefore be interpreted cautiously.

### Sensitivity analyses

Sensitivity analyses excluding Virginia and Utah—states that enacted both sanctuary and anti-sanctuary policies—did not meaningfully alter our estimates. Results were also robust to alternative comparison group specifications, including not-yet-treated counties, with estimates remaining similar in magnitude across models (Appendix Table S2). Additional sensitivity analyses using the primary analytic specification, including the baseline propensity score, with the addition of a time-varying indicator of county-level sanctuary policy status yielded directionally consistent findings relative to the primary analyses (Appendix Table S3). Since inclusion of the time-varying county-level sanctuary policy status resulted in insufficient group-time-specific variation for the doubly robust estimator, these models were estimated using the regression-adjustment estimator. Additional overall (non-stratified) models using the primary analytic specification and additionally adjusting for time-varying immigration enforcement and county-level sanctuary policy status also produced directionally consistent findings (Appendix Table S4). Secondary voting analyses also showed no meaningful association between state sanctuary policy enactment and registered voter turnout (Appendix Figure S4, Appendix Table S5).

## Discussion

In this national, longitudinal analysis, we found no statistically significant association between state sanctuary policy enactment and county-level SNAP and Medicaid participation. However, estimated effects were consistently positive, particularly in counties with high immigration enforcement. The small number of treated states (*n* = 12) likely reduced statistical precision, resulting in wide confidence intervals that warrant cautious interpretation.

Importantly, these outcomes capture effects on the authorized immigrant, lawfully permanent resident, and US-born populations within counties. Because individuals targeted by immigration enforcement are generally ineligible for federal safety-net programs, these findings reflect spillover effects among eligible populations (eg, US citizens and authorized immigrants) whose program participation and healthcare utilization may be influenced by enforcement climates. Stratification by baseline enforcement provides additional context for interpreting these findings. Counties with higher enforcement activity may proxy environments with greater exposure to immigration enforcement, including higher concentrations of unauthorized immigrants or individuals socially connected to them. In these settings, sanctuary policies may have a greater potential to influence program participation by reducing perceived risk and institutional distrust.

Our findings should be interpreted in the context of our previous work. In a prior national county-level study, we found that higher levels of local immigration enforcement, measured using ICE detainer requests, were associated with lower Medicaid and SNAP participation among likely eligible adults.^8^ Building on those findings, the present study evaluates a related but different question: whether state sanctuary policy enactment—a policy intended to limit cooperation with federal immigration authorities—is associated with changes in program participation over time. While our earlier study identified associations between local enforcement and program participation, this study leverages a staggered difference-in-difference design to strengthen causal inference by accounting for differential policy timing, secular trends, and stable differences between treated and comparison states.

Although not statistically significant, the estimated effects were directionally consistent with higher estimated Medicaid and SNAP participation following sanctuary policy enactment. Unlike our previous study, however, these estimates did not reach statistical significance. Rather than contradicting our previous conclusions, these findings refine them by suggesting that any effect of sanctuary policies’ enforcement-related chilling effects remains uncertain. The small number of treated states likely reduced statistical precision, contributing to wider confidence intervals. Together, these findings suggest that additional evaluations of sanctuary policies are needed before drawing firm conclusions regarding their causal effects on public program participation.

State sanctuary policies that limit cooperation between local law enforcement and federal immigration authorities may reduce perceived enforcement risk,^27-29^ providing insight into one mechanism through which immigration policies influence engagement with public institutions. Immigration enforcement may have spillover effects beyond individuals directly targeted by enforcement. Eligible members of mixed-status households and individuals socially proximal to immigrants may avoid healthcare or public benefit programs out of concern that interactions with public institutions could expose themselves or others to immigration enforcement.^7,8^

Our findings also highlight the importance of considering both formal and informal mechanisms through which immigration policies shape behavior. Beyond limiting cooperation between local law enforcement and federal immigration authorities, sanctuary policies often include welcoming or anti-discriminatory provisions that are intended to support the integration of immigrants and may expand access to state and local services that facilitate participation in social institutions.^30,31^ Consequently, sanctuary policies could influence public program participation not only by reducing exposure to immigration enforcement but also by signaling institutional support and fostering trust in public institutions.^31^ Emerging policy feedback research similarly suggests that immigrant-inclusive local policies can increase civic efficacy and engagement by changing how immigrants perceive and interact with public institutions. These findings support the idea that policy environments shape behavior through mechanisms extending beyond formal legal protections, which provides a complementary framework for understanding how sanctuary policies may affect participation in public programs.^13^ Qualitative studies indicate that living in sanctuary jurisdictions may reduce, but not fully eliminate, fear and distrust because federal immigration enforcement continues to occur and uncertainty surrounding immigration policy persists.^32^ Perceptions of safety, trust, and risk are not only consequential for immigrants but also for members of mixed-status households and individuals socially proximal to immigrants, who may similarly avoid healthcare and public programs out of fear of immigration enforcement.^32,33^ This may help explain the generally positive but imprecise associations observed in our study, particularly if sanctuary policies reduce fear for some individuals while broader federal immigration enforcement continues to discourage participation among others.^32^ Together, these findings suggest that sanctuary policies alone may be insufficient to overcome enforcement-related chilling effects. Their effectiveness may depend not only on policy adoption but also on implementation and whether they meaningfully change community perceptions of institutional trust and safety. Future research should examine whether awareness of sanctuary policies mediates their effects on health and social outcomes.

## Conclusion

Although we observed no statistically significant associations between state sanctuary policy enactment and public program participation, estimated effects were consistently positive and aligned with the hypothesis that sanctuary policies may reduce enforcement-related chilling effects. However, given the imprecision of the estimates, these findings should be interpreted cautiously and confirmed in future studies with greater statistical power.

These findings are particularly relevant as federal actions have simultaneously expanded immigration enforcement while proposing reductions to Medicaid and SNAP. Together, these changes may increase barriers to healthcare and public benefit participation among eligible households living in communities affected by immigration enforcement. While sanctuary policies may help mitigate enforcement-related chilling effects, legal protections alone may be insufficient if individuals remain unaware of these policies or continue to distrust public institutions. As policymakers consider future immigration and safety-net reforms, efforts to reduce enforcement-related barriers and strengthen trust in public institutions may be critical to ensuring eligible households are not discouraged from accessing health and nutrition supports.

## Contribution statement

A.E.—conceptualization, methodology, data curation, analysis, writing—original draft, writing—editing and reviewing; C.K.—conceptualization, methodology, writing—editing and reviewing, funding acquisition; A.S-M.—conceptualization, methodology, writing—editing and reviewing, funding acquisition; T.R.—methodology, analysis, writing—editing and reviewing; S.L.L.—data curation, writing—editing and reviewing; B.L—conceptualization, methodology, writing—editing and reviewing, funding acquisition

## Supplementary Material

qxag212_Supplementary_Data
