# Supplementary material for: State sanctuary policies and the immigration enforcement activities across US counties: implications for population health
Source: Health Aff Sch. 2026 Aug 14;4(8):qxag212. doi: 10.1093/haschl/qxag212 (PMC13505508; doi:10.1093/haschl/qxag212)
Supplement: Supplementary file 1 [file qxag212_supplementary_data.zip › Appendix_final.docx]

# **Appendix A. Research Protocol for State Sanctuary Policy Dataset**

# **Research Protocol**

I. **Date of Protocol:** September 2025
II. **Scope:** This longitudinal dataset includes state- and local-level sanctuary and anti-sanctuary policies that determine the extent to which law enforcement officials assist federal immigration authorities in carrying out immigration enforcement activities as well as captures elements of those policies related to select public health outcomes. We included state policies in our dataset that were enacted between 2009-2021. Specifically, our dataset includes:
    a. **State laws** that preempt (limit) local government’s authority to regulate the above
    b. **State laws** that cut funding or impose a financial penalty for local government that enact such laws

Rationale: Due to resource limitations, we are unable to validly measure all laws that could potentially affect immigrants. In this dataset we are focused on immigration enforcement-related laws and policies, described below in our inclusion criteria, that either attempt to ensure immigrants are not deported or reduce the threat of immigration enforcement when they come into contact with the criminal justice system, or, contrastingly, that increase the ability of local and state law enforcement to deport and surveil immigrants.

III. **Primary data collection**
     a. **Project Dates:** June 2024-December 2025
     b. **Dates Covered in the Dataset:** This dataset includes state sanctuary or anti-sanctuary policies enacted between 2009-2021 as well as local sanctuary policies and 287g agreements enacted between 2000-2021. The dataset does not reflect any amendments to any of the policies nor does it include any end dates for state policies and local sanctuary policies. This is because policy end dates were not readily available in the search engines we employed. Therefore, we assumed that once a policy became effective, it remained in effect through the duration of our study period. However, the dataset does include end dates for local 287(g) agreements because these agreements have a specified expiration date. Justification for our study years is explained in detail below.
     c. **Inclusion criteria** – **We are including the following types of laws:**
        i. State sanctuary policies – state-level policies enacted between 2009-2021 which limit the authority of local law enforcement officers (officers in general, not a specific category of law enforcement) to cooperate with federal immigration authorities or carry out immigration-related activities, or which limit cooperation in *civil* investigations to support community policing goals.
     d. **Exclusion criteria** – **We are excluding the following types of laws:**
        i. Federal laws
        ii. Statutes or regulations that address immigration but not criminal justice/law enforcement
        iii. Statutes or regulations that only specify authority of particular categories of law enforcement (e.g., school resource officers, juvenile corrections officers)
        iv. Jail facility contracts (i.e., contracts that allow federal immigration authorities to place detainees in state or local facilities when there is no more room at federal detention centers in the area)
        v. Case law
        vi. State-level laws enacted outside of 2009-2021
     e. **Data Collection Methods:**
        i. State sanctuary policies:
           1. Started with the seminal article, “Understanding Sanctuary Cities”^4^ which has an appendix with links to legislative documentation of several hundred sanctuary policies across the U.S. We identified policies in this appendix that met inclusion criteria.
           2. Searched for additional policies using the NCSL immigration search engine^3^ and search terms ‘status’, ‘sanctuary’, and ‘287’.
           3. Did an additional search on NCSL (searched categories omnibus, education, and law enforcement).
           4. Policies were included from NCSL if enacted between 2009-2021 and included information on local law enforcement officers and limiting cooperation with federal immigration authorities.
     f. **Total policies included in dataset:**
        i. 25 state sanctuary policies
     g. **Note about dates for state-level policies:**
        i. We screened policies for enacted dates and effective dates.
        ii. Enacted date was defined as the date that the policy was passed in the state House and Senate (this was written into the legislation and often says ‘enacted on [DATE]’).
        iii. For the purposes of inclusion criteria, we used the enacted date (meaning, a state policy was considered within the study period if the enacted date was between 2009 and 2021 for state policies.
        iv. Effective date was defined as the date the policy goes into effect (sometimes, usually in bills, this was written into the legislation as saying ‘goes into effect on [DATE]’ or ‘goes into effect XX days after legislative session’).
            1. If the number of days after session was given, we looked up the dates of the legislative session and counted the number of days to determine the effective date.
            2. If there was nothing in the legislation about effective date, we inferred the effective date.^6^
        v. For the purposes of our empirical analyses we used effective date to determine when a policy exposure started.
        vi. We did not include end dates in our dataset for state-level policies because these dates were not readily available in the legislative text or search engines we used. So, we assumed that once these policies were in effect, they remained in effect.
IV. **Coding State and Local Policies:**
    a. **State sanctuary policies**
       i. Policies were coded by three different coders (different combinations of two coders did each round of coding).
       ii. At each round of coding, we calculated inter-coder reliability.^16^
       iii. We developed a coding instrument via Qualtrics.^18^
       iv. One coder read through four state policies to create the coding instrument. The entire research team discussed the instrument until agreement was reached that it could adequately code sanctuary policies.
       v. Two coders redundantly coded five policies to test instrument; disagreement was discussed between both coders until agreement was reached on any differing answers.
       vi. Two coders redundantly coded four more policies – inter-coder reliability = 0.97; disagreement was discussed between both coders until agreement was reached on any differing answers.
       vii. Two coders coded two more policies (one each) and then redundantly coded a random sample of one state policy (50%) – inter-coder reliability = 0.95; disagreement was discussed between both coders until agreement was reached on any differing answers.
            1. Decided on the policy to redundantly code using the randbetween formula in Excel.
       viii. Two coders coded three more policies and then redundantly coded a random sample of one state policy (33%) – inter-coder reliability = 0.80; disagreement was discussed between both coders until agreement was reached on any differing answers.
             1. Due to the low inter-coder reliability, the two coders randomly selected one of the remaining two policies and redundantly coded – inter-coder reliability = 0.96; disagreement was discussed between both coders until agreement was reached on any differing answers.
       ix. Two coders coded nine more policies and then redundantly coded a random sample of two polices (22%) – inter-coder reliability = 0.87; disagreement was discussed between both coders until agreement was reached on any differing answers.
           1. Due to the low inter-coder reliability, the two coders randomly selected two of the remaining policies and redundantly coded – inter-coder reliability = 0.87; disagreement was discussed between both coders until agreement was reached on any differing answers.
       x. Then, we followed the same translating process and scale as the anti-sanctuary policies, but with the following nine elements of interest:
          1. …Complying with ICE detainer requests
          2. …Ban on 287(g) agreements (coded as 0 or 1 only)
          3. …Ban on use of public resources for immigration enforcement (coded as 0 or 1 only)
          4. …Asking about immigration status during routine law enforcement activities (e.g., during traffic stops)
          5. …Asking victims and witnesses of a crime about their immigration status
          6. …Communicating with federal immigration authorities
          7. …Allowing ICE access to detainees or detention facilities
          8. …Discriminating in access to public services or benefits based on immigration status
          9. …Specific law enforcement populations participating in immigration enforcement (e.g., school resource officers)
V. **References**
   1. 1.	ICE's 287(g) Program. United States Department of Homeland Security Accessed July 31, 2023, 2023. <https://www.ice.gov/identify-and-arrest/287g>
   2. 2.	U.S. Immigration and Customs Enforcement. 287(g) - Memorandums of Agreement/Understanding (Old). Department of Homeland Security. <https://www.ice.gov/identify-and-arrest/287g/287g-archive>
   3. 3.	National Conference of State Legislatures. Immigration Legislation Archived Database | 2008-2023. 2024-2025. <https://www.ncsl.org/immigration/immigration-legislation-archived-database>
   4. 4.	Lasch CN, Chan RL, Eagly IV, et al. Understanding Sanctuary Cities. *Boston College Law Review*. 2018;
   5. 5.	The 287(g) Program: An Overview. *American Immigration Council* blog. <https://www.americanimmigrationcouncil.org/fact-sheet/287g-program-immigration/>
   6. files/727/287g-program-immigration.html
   7. 6.	Temple University Center for Public Health Law Research. *Effective Dates*. Vol. 2024. 2022. <https://phlr.org/sites/default/files/uploaded_images/Effective%20Dates.pdf>
   8. 7.	Town of New Paltz Town Board. *Notice of Adoption (Immigraton and Detention Local Law)*. 2017. <https://www.townofnewpaltz.org/sites/g/files/vyhlif3541/f/agenda/agenda-file/6a-2._immigration_detention_ll_notice_of_adoption_04_17_17.pdf>
   9. 8.	Liu P. New Paltz declared a 'sanctuary' for immigrants. *Times Herald-Record*. <https://www.recordonline.com/story/news/2017/05/07/new-paltz-declared-sanctuary-for/21098307007/>
   10. 9.	Secretary Napolitano Announces New Agreement for State and Local Immigration Enforcement Partnerships & Adds 11 New Agreements. 2009. <https://www.dhs.gov/archive/news/2009/07/10/secretary-announces-new-agreement-state-and-local-immigration-enforcement>
   11. 10.	Ballvé M. Wrong Arm of the Law. *Reimagine RP&E*. <https://www.reimaginerpe.org/node/3569>
   12. 11.	New Jersey Advisory Committee to the U.S. Committee on Civil Rights. *Overcoming the Barriers Faced by Immigrants*. 2010. <https://www.usccr.gov/files/pubs/docs/09-30-10_NJSACRptonImmigration.pdf>
   13. 12.	Duffy J. New Jersey's Latino Leadership asks Obama to Rescind Morristown's 287(g) Status. *NewJerseycom*. <https://www.nj.com/morristown/jamieduffy/2009/07/new_jerseys_latino_leadership.html>
   14. 13.	Drobness T. Outgoing mayor enrolls Morristown into immigration program to deputize officers. *NewJerseycom*. <https://www.nj.com/news/2009/12/outgoing_mayor_enrolls_morrist.html>
   15. 14.	The 287(g) Program: State and Local Immigration Enforcement (Congressional Research Service) (2021).
   16. 15.	U.S. Immigration and Customs Enforcement Office of State and Local Coordination. Delegation of Immigration Authority Section 287(g) Immigration and Nationality Act. <https://web.archive.org/web/20100423184138/http:/www.ice.gov/pi/news/factsheets/section287_g.htm>
   17. 16.	Center for Public Health Law Research. *How to Calculate the Rate of Divergence*. 2022. <https://phlr.org/sites/default/files/uploaded_images/Calculating%20a%20Divergence%20Rate.pdf>
   18. 17.	State Anti-Sanctuary Qualtrics Instrument. <https://nam10.safelinks.protection.outlook.com/?url=https%3A%2F%2Fdrexel.qualtrics.com%2Fjfe%2Fform%2FSV_3fJyvtjxk3CGmIS&data=05%7C02%7Cck975%40drexel.edu%7C8e5ae3ba892249c2a81908dd512e0541%7C3664e6fa47bd45a696708c4f080f8ca6%7C0%7C0%7C638755980285552433%7CUnknown%7CTWFpbGZsb3d8eyJFbXB0eU1hcGkiOnRydWUsIlYiOiIwLjAuMDAwMCIsIlAiOiJXaW4zMiIsIkFOIjoiTWFpbCIsIldUIjoyfQ%3D%3D%7C0%7C%7C%7C&sdata=oFiz6M%2FKUsuphZmHjuVMeNq4tRmoH7hqvoJIn%2BDk%2Bd0%3D&reserved=0>
   19. 18.	State Sanctuary Qualtrics Instrument. <https://nam10.safelinks.protection.outlook.com/?url=https%3A%2F%2Fdrexel.qualtrics.com%2Fjfe%2Fform%2FSV_1MTFb4155rIT0gu&data=05%7C02%7Cck975%40drexel.edu%7Ce94b159446c14885b08508dcdd7c9e92%7C3664e6fa47bd45a696708c4f080f8ca6%7C0%7C0%7C638628774518708294%7CUnknown%7CTWFpbGZsb3d8eyJWIjoiMC4wLjAwMDAiLCJQIjoiV2luMzIiLCJBTiI6Ik1haWwiLCJXVCI6Mn0%3D%7C0%7C%7C%7C&sdata=i8JEHUnIAEP5r1kq9Oo6j2Sp4UGDfzWTNcuKU8GXqC4%3D&reserved=0>

# **Appendix Figure 1. Inclusion Criteria Flow Charts**

# **Appendix Figure 2. Parallel Trend Plots for Primary and Secondary Outcomes**

|  |  |
| --- | --- |
|  | |

# **Appendix Figure 3. Conditional Dynamic Event Study Plots, Low-Enforcement Samples**

Note: These event study plots are conditionally based on the adjusted covariates. These covariates include baseline indicators of (1) county population proportion non-citizen, (2) a binary indicator of whether the county ever enacted a 287(g) agreement, (3) the political party affiliation of the governor party.

# **Appendix Figure 4. Event Study Plots – Registered Voting (High and Low Enforcement)**

# **Appendix Table 1. Staggered Difference-in-Difference Estimates for Medicaid and SNAP**

| **Caption**: Percent change in outcome prevalence associated with state sanctuary policy enactment, stratified by baseline immigration enforcement | | | | | |
| --- | --- | --- | --- | --- | --- |
|  | Counties with low immigration enforcement ^a^ | |  | Counties with high immigration enforcement ^a^ | |
| **Outcome** | Unadjusted Percent Change in Prevalence (95% CI) | Adjusted Percent Change in Prevalence (95% CI) |  | Unadjusted Percent Change in Prevalence (95% CI) | Adjusted Percent Change in Prevalence (95% CI) |
|  |  |  |  |  |  |
| Medicaid Participation ^b^ (%) | 11.5 (-4.3, 30.0) | 2.8 (-25.1, 41.1) |  | 16.3 (-18.2, 65.6) | 28.6 (-14.9, 94.3) |
| SNAP Participation ^c^ (%) | 24.2 (-57.9, 266.7) | 27.5 (-42.2, 181.2) |  | 11.3 (-60.5, 213.7) | 9.9 (-81.6, 557.5) |
| **NOTES**: these percent change values are derived from the exponentiated ATT estimates from the difference-in-difference model. | | | | | |
| ^a^ Immigration enforcement quantified at baseline (2011) - low enforcement <= median number of detainer requests per 10,000 non-citizens; high enforcement > median number of detainer requests per 10,000 non-citizens. | | | | | |
| ^b^ Medicaid program participation: Estimated by the number of individuals participating in Medicaid divided by the total number of individuals in poverty (less than or equal to 200% FPL) per county and year (per 10,000 likely-eligible low-income individuals) | | | | | |
| ^c^ Supplemental Nutrition Assistance Program (SNAP) participation: Estimated by the number of households participating in SNAP divided by the total number of low-income households per county and year (per 10,000 households) | | | | | |
| **Fully adjusted models included**: (1) baseline county-level proportion of non-citizens, labor force participation, and state governor party affiliation; and a (2) time-invariant covariate, indicating whether county ever had a 287(g) agreement.  Note: all Medicaid models only had one year in the pre-treatment period due to data restrictions in county-level Medicaid participation. | | | | | |

# **Appendix Table 2. Staggered Difference-in-Difference Estimates with Alternative Comparisons (not-yet-treated counties)**

|  | Counties with low immigration enforcement ^a^ |  | Counties with high immigration enforcement ^a^ |
| --- | --- | --- | --- |
| **Outcome** | Adjusted Percent Change in Prevalence (95% CI) |  | Adjusted Percent Change in Prevalence (95% CI) |
| Primary Outcomes |  |  |  |
| Medicaid Participation ^b^ (%) | 2.8 (-24.3, 39.5) |  | 28.6 (-14.2, 92.8) |
| SNAP Participation ^c^ (%) | 28.9 (-35.0, 151.9) |  | 9.3 (-82.1, 568.6) |
| **Note**: these percent change values are derived from the logged ATT estimates from the difference-in-difference model.  These estimates test an alternative comparison group in the difference-in-difference estimates: not-yet-treated counties. | | | |
| ^a^ Immigration enforcement quantified at baseline (2011) - low enforcement <= median number of detainer requests per 10,000 non-citizens; high enforcement > median number of detainer requests per 10,000 non-citizens. | | | |
| ^b^ Medicaid program participation: Estimated by the number of individuals participating in Medicaid divided by the total number of individuals in poverty (less than or equal to 200% FPL) per county and year (per 10,000 likely-eligible low-income individuals) | | | |
| ^c^ Supplemental Nutrition Assistance Program (SNAP) participation: Estimated by the number of households participating in SNAP divided by the total number of low-income households per county and year (per 10,000 households) | | | |
| **Fully adjusted models included**: (1) baseline county-level proportion of non-citizens, labor force participation, and state governor party affiliation; and a (2) time-invariant covariate, indicating whether county ever had a 287(g) agreement.  Note: all Medicaid models only had one year in the pre-treatment period due to data restrictions in county-level Medicaid participation. | | | |

# **Appendix Table 3. Staggered Difference-in-Difference Sensitivity Analyses Adjusting for County-Level Sanctuary Policy Status**

|  | Counties with low immigration enforcement ^a^ |  | Counties with high immigration enforcement ^a^ |
| --- | --- | --- | --- |
| **Outcome** | Adjusted Percent Change in Prevalence (95% CI) |  | Adjusted Percent Change in Prevalence (95% CI) |
| Primary Outcomes |  |  |  |
| Medicaid Participation ^b^ (%) | 9.0 (-3.1, 22.5) |  | 15.1 (-25.9, 78.8) |
| SNAP Participation ^c^ (%) | 33.1 (-30.3, 154.1) |  | 10.7 (-76.6, 424.1) |
| **Note**: Percent changes were derived from the logged average treatment effects on the treated (ATT) estimates from the staggered difference-in-differences models. These sensitivity analyses present stratified models adjusting for time-varying county-level sanctuary policy status. These sensitivity analyses were estimated using the regression-adjustment estimator rather than the doubly robust estimator used in the primary analyses because inclusion of the time-varying county-level sanctuary policy status resulted in a singular post-treatment regression design matrix in the doubly robust specification. | | | |
| ^a^ Immigration enforcement quantified at baseline (2011) - low enforcement <= median number of detainer requests per 10,000 non-citizens; high enforcement > median number of detainer requests per 10,000 non-citizens. | | | |
| ^b^ Medicaid program participation: Estimated by the number of individuals participating in Medicaid divided by the total number of individuals in poverty (less than or equal to 200% FPL) per county and year (per 10,000 likely-eligible low-income individuals) | | | |
| ^c^ Supplemental Nutrition Assistance Program (SNAP) participation: Estimated by the number of households participating in SNAP divided by the total number of low-income households per county and year (per 10,000 households) | | | |
| **Fully adjusted models included**: (1) baseline county-level proportion of non-citizens, labor force participation, and state governor party affiliation; (2) time-invariant covariate, indicating whether county ever had a 287(g) agreement; and (3) time-varying indicator of county-level sanctuary policy status.  All Medicaid models only had one year in the pre-treatment period due to data restrictions in county-level Medicaid participation. | | | |

# **Appendix Table 4. Sensitivity Analyses: Overall Staggered Difference-in-Differences Models**

| **Outcome** | **Adjusted Percent Change in Prevalence (95% CI)** | | |
| --- | --- | --- | --- |
| Primary Outcomes |  |  |  |
| Medicaid Participation ^a^ (%) | 9.3 (-5.3, 26.1) | | |
| SNAP Participation ^b^ (%) | 11.2 (-8.5, 35.3) | | |
| **Note**: Percent changes were derived from the logged average treatment effects on the treated (ATT) estimates from the staggered difference-in-differences models. These sensitivity analyses present stratified models adjusting for time-varying county-level sanctuary policy status and a binary indicator of high versus low immigration enforcement (at baseline). These sensitivity analyses were estimated using the regression-adjustment estimator rather than the doubly robust estimator used in the primary analyses because inclusion of the time-varying county-level sanctuary policy status resulted in a singular post-treatment regression design matrix in the doubly robust specification. | | | |
| ^a^ Medicaid program participation: Estimated by the number of individuals participating in Medicaid divided by the total number of individuals in poverty (less than or equal to 200% FPL) per county and year (per 10,000 likely-eligible low-income individuals) | | | |
| ^b^ Supplemental Nutrition Assistance Program (SNAP) participation: Estimated by the number of households participating in SNAP divided by the total number of low-income households per county and year (per 10,000 households) | | | |
| **Fully adjusted models included**: (1) baseline county-level proportion of non-citizens, labor force participation, immigration enforcement (low vs. high), and state governor party affiliation; (2) time-invariant covariate, indicating whether county ever had a 287(g) agreement; and (3) time-varying indicator of county-level sanctuary policy status. | | | |

# **Appendix 5. Staggered Difference-in-Difference Estimates for Registered Voter Turnout**

|  | Counties with low immigration enforcement ^a^ | |  | Counties with high immigration enforcement ^a^ | |  |
| --- | --- | --- | --- | --- | --- | --- |
| **Outcome** | Unadjusted Percent Change in Prevalence (95% CI) | Adjusted Percent Change in Prevalence (95% CI) |  | Unadjusted Percent Change in Prevalence (95% CI) | Adjusted Percent Change in Prevalence (95% CI) |  |
| Secondary Outcome |  |  |  |  |  |  |
| Voting participation (%) | -3.84  (-34.40, 40.95) | -2.74  (-27.95, 31.28) |  | -8.61  (-28.53, 16.86) | -12.74 (-28.01, 5.77) |  |
| ^a^ Immigration enforcement quantified at baseline (2011) - low enforcement <= median number of detainer requests per 10,000 non-citizens; high enforcement > median number of detainer requests per 10,000 non-citizens. | | | | | |  |
| **Fully adjusted models included**: (1) time-varying covariates, including county-level proportion of non-citizens, labor force participation, and state governor party affiliation; and a (2) time-invariant covariate, indicating whether county ever had a 287(g) agreement. | | | | | |  |

References

1. Burris S. How to write a legal mapping paper. *Temple University Legal Studies Research Paper*. 2020;(2018-10)
2. Burris S. A technical guide for policy surveillance. *Temple University legal studies research paper*. 2014;(2014-34)
